# Minimally Invasive Mitral Valve Surgery in Elderly Patients: Results from a Multicenter Study

**DOI:** 10.3390/jcm13216320

**Published:** 2024-10-23

**Authors:** Alessandra Francica, Cristina Barbero, Filippo Tonelli, Alfredo Giuseppe Cerillo, Vittoria Lodo, Paolo Centofanti, Giovanni Marchetto, Germano Di Credico, Ruggero De Paulis, Pierluigi Stefano, Giovanni Battista Luciani, Francesco Onorati, Mauro Rinaldi

**Affiliations:** 1 Department of Cardiac Surgery, University of Verona, 37126 Verona, Italy; alessandrafrancica@yahoo.it (A.F.); filippo.tonelli@univr.it (F.T.); giovannibattista.luciani@univr.it (G.B.L.); francesco.onorati@univr.it (F.O.); 2 Cardiac Surgery Unit, Citta della Salute e della Scienza, 10126 Torino, Italy; mauro.rinaldi@unito.it; 3 Cardiac Surgery Unit, Careggi University Hospital, 50134 Firenze, Italy; acerillo@yahoo.com (A.G.C.); pierluigi.stefano@unifi.it (P.S.); 4 Cardiac Surgery Unit, Azienda Ospedaliera Mauriziano, 10128 Torino, Italy; vittoria.lodo90@gmail.com (V.L.); centofantipaolo@gmail.com (P.C.); 5 Cardiac Surgery Unit, IRCCS San Gerardo dei Tintori, 20900 Monza, Italy; giovanni.marchetto.cch@gmail.com; 6 Cardiac Surgery Unit, ASST Ovest Milanese, 20025 Legnano, Italy; germano.dicredico@asst-ovestmi.it; 7 Cardiac Surgery Unit, European Hospital of Rome, 00149 Roma, Italy

**Keywords:** mitral valve surgery, minimally invasive access, mini-thoracotomy, elderly

## Abstract

**Background:** Minimally invasive mitral valve surgery (MIMVS) has been increasingly adopted worldwide as an alternative to conventional sternotomy, especially for young patients. The remarkable results gained by MIMVS have encouraged its application in more complex and fragile patients, such as the elderly, though results in this subgroup remain controversial. It is the aim of this study to assess the postoperative outcomes of patients older than 75 years old undergoing MIMVS, and to compare these results to those of younger patients. **Methods:** The data of all patients undergoing MIMVS between 2015 and 2022 were retrospectively collected at seven high-volume cardiac surgery centers. Patients were divided into two age-based groups: the young (<65 years old) and the elderly (>75 years old). A propensity score (PS) matching analysis obtained two comparable groups. Postoperative outcomes were assessed in both the unmatched and PS-matched populations. **Results:** Out of 1113 patients undergoing MIMVS, 524 were young and 279 were elderly. Elderly patients were more commonly affected by multiple comorbidities, with a higher EuroSCORE II (4.6 ± 5.5% vs. 1.6 ± 3.3%, *p* < 0.001). There was no difference in postoperative mortality, though the elderly had a greater incidence of postoperative complications, such as re-exploration for bleeding, stroke, reintubation, and a need for hemodialysis and blood transfusions. After PS matching, 119 pairs of young and elderly patients with similar risk profiles (EuroSCORE II 2.5 ± 4.7% vs. 2.7 ± 3.2%, *p* = 0.7) were compared, and no differences in all postoperative outcomes were found. **Conclusions:** Adequately selected elderly patients can report hospital outcomes similar to young patients after MIMVS.

## 1. Introduction

In recent decades, excellent outcomes in patients undergoing minimally invasive mitral valve surgery (MIMVS) have been reported [1,2,3]. Several studies have shown how the less invasive approach has become feasible and effective in mitral valve surgery for both replacement and repair techniques [1,2,3,4,5,6]. MIMVS has proven to be superior to conventional sternotomy in terms of faster healing, improved cosmesis, and shorter hospitalization [4,5,6]. However, these studies have mostly included young patients with low preoperative risk profiles and few comorbidities. Although the incidence of mitral valve disease is reported to be more than 10% in patients older than 70 years old [7], very limited MIMVS experiences have been reported in the elderly, possibly because these patients are universally recognized as fragile and at higher surgical risk [8,9]. Indeed, thanks to acquired expertise and to the improvement in surgical techniques, in recent years, MIMVS has also become the first choice for subgroups who were deemed unsuitable before [10]. To date, few studies have been carried out on the elderly population, and the current outcome of MIMVS in this subgroup remains unclear. Therefore, it is the aim of this large-scale multicenter study to analyze current postoperative results of elderly patients undergoing MIMVS.

## 2. Methods

### 2.1. Study Population and Protocol

From January 2015 to December 2022, the data of consecutive patients undergoing MIMVS were prospectively collected at seven high-volume cardiac surgery centers, and retrospectively reviewed. Both mitral valve replacement and repair were considered, as well as combined procedures (i.e., tricuspid valve surgery, atrial fibrillation surgical ablation, and atrial septal defect closure). Surgical indications for surgery were performed according to the most recent guidelines for the management of valvular heart disease [11]. The exclusion criteria were an age lower than 18 years and salvage procedures. Only surgeons with at least 50 minimally invasive cases per year, and with at least 3 years of experience in minimally invasive cardiac surgery, were able to take part in the study.

In order to analyze the two populations with a significant difference in preoperative risk profiles, two study groups were identified: the young, defined as patients younger than 65 years old, and the elderly, defined as patients older than 75 years. The age cut-offs were defined according to the latest ESC/EACTS Guidelines on heart valve diseases [11]. This study was conducted according to the guidelines of the Declaration of Helsinki and approved by the institutional review board of each center (date of approval in the pilot center: 19 October 2022; protocol number: 0001455; University of Turin). Informed consent was obtained from all subjects involved in this study.

The primary endpoint of this study was postoperative mortality. The secondary endpoints were stroke, re-exploration for bleeding, conversion to sternotomy, respiratory failure with reintubation, a need for blood transfusion, the new onset of atrial fibrillation or conduction disturbances requiring pacemaker implantation [12], and acute kidney injury [13].

Postoperative mortality was defined as death occurring 30 days after the index procedure or >30 days during the index hospitalization, according to the latest Valve Academic Research Consortium 3 (VARC-3) criteria [12]. Stroke was defined as clinical signs persisting at the time of discharge from the hospital and/or in the presence of localized ischemic infarcts detectable by conventional neuroimaging techniques. Conservative surgery on the mitral valve was defined as simple in cases of P2 prolapse repair with resection or chords; it was defined as complex in all the other cases.

### 2.2. Surgical Technique

The surgical approach, perfusion strategies, and aortic clamping techniques used for patients undergoing MIMVS have already been described [14,15]. Briefly, all patients underwent operation through a right mini-thoracotomy in the fourth intercostal space and with a double-lumen endotracheal tube to allow single-lung ventilation. A soft tissue retractor was used to expose the surgical port, and an endoscope was inserted in an accessory port created below the working port; the same port was used for carbon dioxide insufflation. An additional a sixth intercostal space port was created for pump suction. After full heparinization, a peripheral cardiopulmonary bypass was established with the patient cooled to 30–32 °C. The arterial perfusion strategies and aortic clamping techniques used during the study period were retrograde arterial perfusion (RAP) with an endo-aortic clamp, RAP with a trans-thoracic clamp, RAP with a beating heart, and antegrade arterial perfusion through the axillary artery with a trans-thoracic clamp. The former was usually preferred in cases of previous cardiac surgery procedures, while the latter was usually preferred to provide antegrade systemic perfusion in cases of severe atherosclerotic burden. The choice of one setting in respect to the others was, in part, patient-orientated (anatomy and clinical history) and, in part, dependent on the availability of the different settings at different periods. Venous return was routinely obtained with a double cannulation (jugular and femoral). All cannulas were inserted with the Seldinger technique, either under direct vision in cases of vascular surgical exposure or percutaneously.

In the endo-aortic clamping setting, aortic occlusion and cardioplegia delivery were performed with a balloon catheter inserted through the sidearm of a femoral arterial cannula (21F or 23F Intraclude, Edwards Lifesciences, Irvine, CA, USA). In the trans-thoracic clamping setting, the clamp was directed towards the ascending aorta through the first intercostal space with a Chitwood clamp or through the main port with a Cygnet flexible clamp. Cardioplegia was delivered with a 7F cardioplegia needle (CalMed Technologies, Santa Inez, CA, USA) placed into the proximal ascending aorta. Antegrade myocardial protection was provided with St. Thomas (Plegisol, Hospira Inc., Lake Forest, IL, USA) or Custodiol (Bretschneider histidine, tryptophan, ketoglutarate solution, Kohler Chemie, Bensheim, Germany) cold crystalloid cardioplegia. Superior and inferior vena cava snaring was performed by placing tourniquets around the vessels or by placing endovascular balloons to provide a temporary mini right atriotomy to drain the cardioplegic solution and in patients requiring associated right atrial procedures, most commonly tricuspid valve repair. The valve was exposed through a standard left atriotomy, parallel and posterior to the interatrial septum. Clamp release was performed at a core temperature above 33 °C during rewarming. Intraoperative transesophageal echocardiography is mandatory and was used in all patients to guide the correct positioning of the cannulas before the onset of cardiopulmonary bypass and to assess cardiac function, residual mitral valve regurgitation in case of repair, paravalvular leaks, and prosthetic gradients after the intracardiac phase of the operation.

### 2.3. Statistical Analysis

Descriptive statistics were used to analyze the data. Continuous data are reported as means ± standard deviation and categorical variables are reported as counts and percentages. Differences between unmatched groups were assessed using Student’s *t*-test and Chi-square test or Fisher’s exact test, as appropriate. To balance the distribution of baseline risk factors between groups, propensity score (PS) matching was performed using a multivariable logistic regression model, for which the independent variables were the statistically significant preoperative differences between the two groups. A nearest matching algorithm was used with a caliper of 0.01. A standardized mean difference of <0.1 (10%) was used to assess the balance between the two PS-matched groups. The thirty-day outcomes in the matched groups were then assessed. The statistical analysis was performed using SPSS Version 27.0 (IBM Corp., Armonk, NY, USA). A *p*-value of <0.05 was considered statistically significant.

## 3. Results

**Figure 1:**
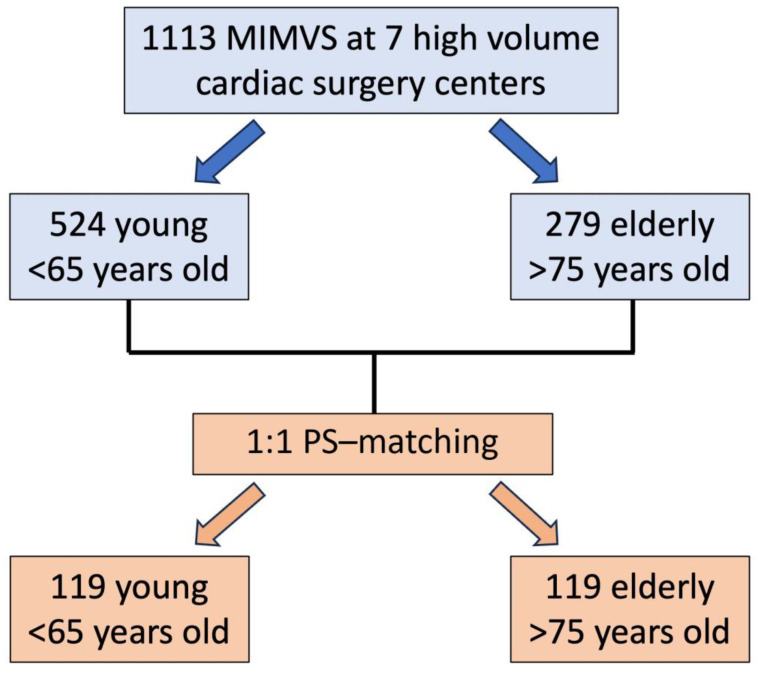
Flowchart of this study.

During the study period, 1113 patients underwent MIMVS. Among these, 524 patients were young (53.41 ± 9.7 years old) and 279 were old (78.7 ± 2.7 years old) (Figure 1). Elderly patients were more commonly females and affected by several comorbidities, such as diabetes mellitus, chronic lung disease, pulmonary hypertension, chronic kidney disease, peripheral vascular disease, atrial fibrillation, and concomitant moderate-to-severe tricuspid valve regurgitation, and therefore reported a higher EuroSCORE II (4.6 ± 5.5% vs. 1.6 ± 3.3%; *p* < 0.001) (Table 1). The rates of previous cardiac surgery procedures were comparable between groups: 39 young patients (7.4%) and 25 elderly patients (9%), *p* 0.45.

Femoral artery cannulation was the favorite way to establish cardiopulmonary bypass (CBP) in both groups, although it was slightly less used in elderly patients (97.5% vs. 84.6%; *p* < 0.001). Axillary and direct ascending aortic cannulation were performed more commonly in the elderly (Supplementary Table S1). Endo-aortic balloon was the preferred clamping technique in the younger group (34.7% vs. 11.5%; *p* < 0.001). Concomitant procedures such as tricuspid valve surgery (16.9% vs. 5.2%; *p* < 0.001) or atrial fibrillation surgical ablation (10.4% vs. 6.2%; *p* = 0.002) were more often performed in the elderly, whereas young patients more frequently underwent isolated mitral valve surgery (86.2% vs. 72.8%; *p* < 0.001). Also, repair of the valve was more frequent in the young group (75.9% vs. 46.6%; *p* < 0.001). The intraoperative details are reported in Supplementary Table S1. There was no difference in postoperative mortality between the two populations, though the elderly had a higher incidence of postoperative complications, such as stroke (6.1% vs. 1.3%; *p* < 0.001), conversion to sternotomy (3.9% vs. 1.5%; *p* = 0.03), reintubation (5.1% vs. 2.3%; *p* = 0.04), a need for hemodialysis (4% vs. 1.2%; *p* = 0.009), blood transfusions (40.4% vs. 27.8%; *p* < 0.001), and pacemaker implantation (8.7% vs. 1.2%; *p* < 0.001) (Supplementary Table S2). No differences between groups were reported in terms of postoperative bleeding requiring re-exploration, or new onset of atrial fibrillation.

After PS matching, 119 pairs of young (mean age 53.8 ± 10.5) and elderly patients (mean age 78.5 ± 2.8) (Figure 1) with similar risk profiles (EuroSCORE II 2.5 ± 4.7% vs. 2.7 ± 3.2%) were selected and compared (Table 1). Femoral artery cannulation remained the preferred setting for CBP in both groups, although axillary and direct ascending aortic cannulation were still more used in older patients (axillary: 9.2% vs. 1.7% and ascending aorta: 4.2% vs. 0.8%; *p* < 0.001). Similarly, endo-aortic clamping remained a favorite option for young patients (34.7% vs. 11.5%; *p* < 0.001). However, after PS matching, there were no more differences in terms of mitral valve surgery: repair and replacement was found to be performed equally in the two groups, as were combined procedures. The intraoperative details are reported in Table 2. The two PS-matched populations showed no differences in postoperative outcomes, with low rates of stroke, reintubation, need for hemodialysis, blood transfusions, and pacemaker implantation. The postoperative outcomes are displayed in Table 3.

## 4. Discussion

The present multicenter study demonstrates that preoperative comorbidities and different risk profiles between young and elderly patients can explain the traditionally reported higher incidence of postoperative complications in patients older than 75 years old. When the preoperative risk profiles are similar, the hospital outcomes become comparable between the two groups. Surprisingly, postoperative mortality was very low in both the unmatched and matched populations, and this could be explained by the time-period of the study, since it covers the last few years of minimally invasive surgical practice of highly experienced surgeons at seven high-volume Italian cardiac surgery centers, probably excluding the early years of surgical experience and their related learning curves. It is well known, indeed, that the minimally invasive technique requires a longer learning curve to achieve similar mortality and morbidity of conventional approaches [16,17,18]. The good results obtained can also be explained by considering that all patients during the study period underwent a full pre-operative vascular screening with an angio-CT scan or angiography of the aorto-iliac–femoral vessels in order to detect major contraindications to the minimally invasive approach and in order to identify the safest perfusion strategy and aortic clamping technique: RAP with an endo-aortic clamp, RAP with a trans-thoracic clamp, or antegrade arterial perfusion with a trans-thoracic clamp.

Throughout the decades, MIMVS has spread worldwide, since it has been shown to be non-inferior in terms of mortality and superior in terms of recovery from surgery, the rate of wound infection, and intensive care unit and hospitalization length-of-stay compared to standard sternotomy [4,5,6,19]. However, early studies have mainly included young patients [1,2,3,4,5,6], due to the increased mortality and morbidity reported so far in the elderly after cardiac surgery [9]. Recently, thanks to acquired experience and technological improvement, MIMVS has been applied to subgroups of patients previously deemed unsuitable for this approach [10], such as elderly patients. Nevertheless, the current results of MIMVS in elderly patients remain unclear. The published studies also including old patients from the first decade of MIMVS report high postoperative mortality [20,21]: Iribarne et al. [20] reported 7% mortality in patients older than 75 years old undergoing MIMVS; Holzhey et al. [21] found a mortality rate of 7.7% in patients older than 70 years old. For the above-mentioned reasons, both studies do not mirror the current surgical outcome of MIMVS in elderly patients. However, these studies started to demonstrate that older patients undergoing MIMVS had shorter hospitalization, improved postoperative functional status, and low rates of major adverse cardiac or cerebrovascular events when compared to standard sternotomy. Accordingly, a more recent study by Hisatomi et al. [22] reported lower mortality in patients aged > 70 years old, in line with our findings. They also showed that the clinical frailty scale and the occurrence of a major complication were independent negative predictors for early ambulation in the right mini-thoracotomy group, but not for the conservative one [22]. These results support our study, suggesting that concomitant comorbidities are the real factor responsible for illness in the elderly population. Their influence on postoperative patient status was clearly described [23] and then confirmed by Barbero et al. [24], who demonstrated that chronic kidney disease, rather than age, represents a predictive factor for mortality and morbidity in octogenarians undergoing MIMVS. Despite all the above-mentioned studies having investigated the impact of MIMVS in elderly patients, they have always compared MIMVS to conventional sternotomy. Moreover, the age cut-offs chosen are different among studies, and none compare the early postoperative outcomes of elderly patients to those of younger patients, whose benefits after MIMVS are well known. Only a single-center study [25] carried out a similar comparison, between patients >75 years old and those <75 years old: no differences were found in the two PS-matched groups in terms of postoperative mortality and complications, further supporting our findings. However, they reported similar rates of postoperative stroke, but a higher incidence of surgical revision for bleeding, hemodialysis, and 30-day mortality in the elderly when compared to ours results. These findings might be explained by the larger timeline of their study (2011–2021), thus again including the early years of MIMVS experience.

In this subgroup of fragile and high-risk patients, transcatheter valve repair or replacement should be considered as an alternative option to traditional or minimally invasive cardiac surgery [26,27]. However, it is well-known that microinvasive procedures for MV disease have strict parameters of selection, particularly regarding the valve anatomy and relationship with other cardiac structures; therefore, a huge proportion of patients are excluded from receiving the benefits of these options. Moreover, when looking at micro-invasive options for MV replacement, suboptimal outcomes in terms of in-hospital mortality and the weaknesses of these procedures, such as iatrogenic left ventricular outflow tract obstruction and MV stenosis, are still reported and represent a restraint in the broader adoption of these techniques [28,29]. Data from the VIVID (Valve-In-Valve International Data) Registry, on more than 1000 patients from 90 centers undergoing a valve-in-valve or a valve-in-ring procedure, show a 30-day mortality of 7% and a consistent rate of residual stenosis (immediate postprocedural moderate stenosis in 61% of cases, and severe stenosis in 9% of cases) [30]. Therefore, in our opinion, in high-volume heart valve centers with highly experienced surgeons, surgery still remains the standard of care for MV disease high-risk patients.

### 4.1. Limitations

The main limitation of this study stems from its retrospective nature. However, to the best of our knowledge, it represents the first multicenter study to report the current outcomes of elderly versus young patients undergoing MIMVS. Other limitations concern the lack of clinical and echocardiographic follow-up, the lack of details regarding the anatomy of the mitral valve at the time of surgery, and the proportion of resect and respect techniques during valve repair. Certainly, further studies addressing mid-term follow-up of MIMVS in elderly patients are required.

### 4.2. Conclusions

Our study demonstrates that surgery with a minimally invasive approach can provide optimal results even in patients older than 75 years, and that these results are comparable to those reported in younger patients.

## Figures and Tables

**Table 1:** Preoperative characteristics in unmatched and matched populations.

Variables	Young *n*. 524	Elderly *n*. 279	*p*	*SMD*	Young *n*. 119	Elderly *n*. 119	*p*	*SMD*
Males	337 (64.3)	114 (40.9)	<0.001	0.5	67 (56.3)	59 (49.6)	0.3	0.1
BSA, m^2^	1.75 (0.34)	1.6 (0.30)	<0.001	0.4	1.6 (0.33)	1.6 (0.28)	0.5	0.06
EuroSCORE II, %	1.6 (3.11)	4.6 (5.5)	<0.001	0.7	2.5 (4.7)	2.7 (3.2)	0.7	0.05
Cerebrovascular disease	3 (0.6)	6 (2.2)	0.04	0.1	0	1 (0.8)	0.31	0.01
Peripheral vascual disease	17 (3.3)	50 (17.9)	<0.001	0.5	7 (5.9)	17 (14.3)	0.051	0.2
Diabetes mellitus	21(4)	36 (12.9)	<0.001	0.3	5 (4.2)	11 (9.2)	0.2	0.2
Previous stroke	21 (4)	15 (5.4)	0.4	0.05	8 (6.7)	6 (5)	0.6	0.08
Chronic pulmonary disease	31 (5.9)	40 (14.3)	<0.001	0.3	6 (5)	11 (9.2)	0.2	0.1
Liver disease	10 (1.9)	3 (1.1)	0.37	0.06	3(2.5)	2 (1.7)	0.65	0.05
Ejection fraction	61.3 (9.07)	59.6 (9.4)	0.03	0.3	61.3 (10.5)	60.5 (7.9)	0.46	0.05
Creatinine, mg/dL	0.9 (0.52)	1.16 (0.76)	<0.001	0.4	1 (0.68)	1.08 (0.47)	0.25	0.1
Atrial fibrillation	77 (14.7)	138 (49.5)	<0.001	0.9	31 (26.1)	28 (23.5)	0.65	0.06
Moderate/severe TV regurgitation	137 (26.2)	149 (53.6)	<0.001	0.5	47 (39.5)	43 (36.1)	0.59	0.07
sPAP, mmHg	37.7 (13.2)	48.3 (15.3)	<0.001	0.7	40.3 (16.3)	43.5 (14.8)	0.19	0.05
MV regurgitation	483 (92.2)	260 (93.2)	0.8	0.04	103 (86.5)	111 (93.2)	0.42	0.2
MV stenosis	41 (7.8)	19 (6.8)			16 (13.5)	8 (6.8)		

BSA, body surface area; sPAP, systolic pulmonary arterial pressure; MV: mitral valve. Data are expressed as *n* (%) or m (SD) as appropriate.

**Table 2:** PS-matched population: intraoperative variables.

Intraoperative Variables	Young *n*. 119	Elderly *n*. 119	*p* Value
Isolated MV surgery	97 (81.5)	101 (84.9)	0.48
Concomitant TV surgery	10 (8.3)	9 (7.6)	0.18
Concomitant AF surgical ablation	12 (10.1)	5 (4.2)	0.08
Femoral arterial cannulation	116 (97.5)	103 (86.6)	0.008
Axillary arterial cannulation	2 (1.7)	11 (9.2)	
Aortic arterial cannulation	1 (0.8)	5 (4.2)	
Transthoracic aortic clamp	76 (63.9)	113 (95)	<0.001
Endo-aortic clamp	40 (33.6)	6 (5)	<0.001
Simple MV repair	41 (17.2)	52 (21.8)	0.16
Complex MV repair	37 (31.1)	26 (21.8)	
MV replacement	37 (31.1)	40 (16.8)	
Mitral prosthesis replacement	4 (3.4)	1 (0.4)	
CPB time, min	139.2 (36.6)	125 (28.2)	0.002
Clamp time, min	102.68 (32.1)	89.42 (24.7)	0.001
Conversion to sternotomy	1 (0.8)	3 (2.5)	0.31

AF, atrial fibrillation; CPB, cardiopulmonary bypass; MV, mitral valve; TV, tricuspid valve. Data are expressed as n (%) or m (SD) as appropriate.

**Table 3:** PS-matched population: postoperative outcomes.

Postoperative Outcomes	Young *n*. 119	Elderly *n*. 119	*p* Value
ICU days	1.6 (1.6)	2.07(2.3)	0.06
Ventilation time, hours	12.2 (12)	12.6 (11.5)	0.5
Blood transfusion	40 (33.6)	35 (29.7)	0.5
Respiratory failure	5 (4.2)	5 (4.3)	0.9
Stroke	2 (1.7)	5 (4.2)	0.25
Re-exploration for bleeding	3 (2.5)	3 (2.5)	0.6
Hemodyalisis	3 (2.5)	3 (2.5)	0.9
Pacemaker implantation	2 (0.8)	5 (4.2)	0.24
New onset AF	38 (31.9)	49 (41.5)	0.13
30-day mortality	1 (0.9)	0	0.32

AF, atrial fibrillation; ICU, intensive care unit.

## Data Availability

Data is contained within the article or Supplementary Material.

## Supplementary Materials

The following supporting information can be downloaded at: https://www.mdpi.com/article/10.3390/jcm13216320/s1, Table S1: Overall population: intraoperative variables; Table S2: Overall population: post-operative outcomes.
