# Supplementary material for: Minimally Invasive Mitral Valve Surgery in Elderly Patients: Results from a Multicenter Study
Source: J Clin Med. 2024 Oct 23;13(21):6320. doi: 10.3390/jcm13216320 (PMC11545856; doi:10.3390/jcm13216320)
Supplement: Supplementary file 1 [file jcm-13-06320-s001.zip › jcm-3206965-supplementary.pdf]

Supplemental Table S1. Overall population: intraoperative variables

**Intra-operative variables**	**Patients <65 years tot=524** *n(%); m(SD)*	**Patients > 75 years tot=279** *n(%); m(SD)*	***p***
Isolated MV surgery	454 (86.2)	203 (72.8)	*<.001*
Concomitant procedure			
TV surgery	27 (5.1)	47 (16.9)	<.001
AF surgical ablation	32 (6)	28 (10)	.002
Arterial cannulation			
Femoral	509 (97.5)	236 (82.6)	*<.001*
Axillary	9 (1.7)	36 (12.9)	
Aortic	4 (0.8)	7 (2.5)	
Transthoracic Aortic Clamp	337 (64.4)	246 (88.2)	*<.001*
Endo-aortic clamp	187 (35.6)	33 (11.8)	*<.001*
Type of MV surgery			
Simple MV Repair	181 (34.5)	78 (28)	*<.001*
Complex MV Repair	217 (41.4)	52 (18.6)	
MV Replacement	114 (21.8)	141 (50.5)	
Mitral Prosthesis Replacement	12 (2.3)	8 (2.9)	
CPB time, min	132. 9 (34.6)	130,78 (41.1)	*0.46*
Clamp time, min	99.6 (29.4)	91.24 (26.5)	*<.001*
Conversion in Sternotomy	8 (1.5)	11 (3.9)	*0.03*

AF, Atrial Fibrillation; CPB, Cardiopulmonary By-pass; MV, Mitral Valve; TV, Tricuspid Valve

Supplemental Table S2. Overall population: post-operative outcomes

**Post-operative outcomes**	**Patients <65 years tot=524** *n(%); m(SD)*	**Patients > 75 years tot=279** *n(%); m(SD)*	***p***
ICU days	1.68 (4.7)	2.5 (3.5)	*0.015*
Ventilation time, hours	11.9 (11.8)	17.7 (19.9)	*<.001*
Blood transfusion	145 (27.8)	112 (40.4)	*<.001*
Respiratory failure	12 (2.3)	14 (5.1)	*0.04*
Stroke	7 (1.3)	17(6.1)	*<.001*
Surgical revision for bleeding	10 (1.9)	7 (2.5)	*0.8*
Haemodyalisis	6 (1.2)	11 (4)	*.009*
Pace-maker Implantation	6 (1.2)	24 (8.7)	*<.001*
New onset AF	130 (24.8)	100 (36.1)	*<.001*
30-day mortality	3 (0.6)	0	*0.2*

AF, Atrial fibrillation; ICU, Intensive Care Unit
